# Encapsulation of felty germander (*Teucrium polium* L.) extract using the freeze‐drying method

**DOI:** 10.1002/fsn3.4136

**Published:** 2024-05-06

**Authors:** Sima Zeidvand, Sara Movahhed, Hossein Ahmadi Chenarbon, Peyman Rajaei

**Affiliations:** ^1^ Department of Food Science and Technology, College of Agriculture, Varamin – Pishva Branch Islamic Azad University Varamin Iran; ^2^ Department of Agronomy, College of Agriculture, Varamin ‐ Pishva Branch Islamic Azad University Varamin Iran

**Keywords:** encapsulation, Felty germander extract, freeze dryer, Maltodextrin

## Abstract

Extracts with antimicrobial and antioxidant properties are limited in their application in food products due to their inability to withstand harsh environmental conditions, such as high temperatures and oxygen exposure. Therefore, the present study investigated the nanoencapsulation of *Teucrium polium* L. extract using the freeze‐drying method to facilitate its application and protection against environmental factors. In this regard, an emulsion containing *Teucrium polium* L. extract at concentrations of 10%, 20%, and 30% and a mixture of maltodextrin/Persian gum in three ratios of 1:2, 1:1, and 2:1 as the coating wall were produced and then dried in a freeze dryer. In the following, the properties of emulsions and produced nanocapsules were studied. According to the results, emulsions with high amounts of Persian gum showed more stability, zeta potential, and viscosity. However, their particle size and polydisparity index were lower than those of other emulsions. As the extract concentration increased, there was a decrease in stability, zeta potential, and viscosity, accompanied by an increase in particle size and polydispersity index. Concurrently, elevated concentrations of maltodextrin, Persian gum, and extract resulted in higher humidity, density, encapsulation efficiency, and antioxidant activity of the capsules. The most optimal properties of emulsions and nanocapsules were achieved at the 10% concentration of *Teucrium polium* L. extract and the 1:1 ratio of maltodextrin/Persian gum mixture as the wall material. It is noteworthy that the release rate of phenolic compounds reached its maximum value (88%) after 60 days.

## Graphical abstract

An emulsion containing *Teucrium polium* L. extract at concentrations of 10, 20, and 30%, and a mixture of maltodextrin/Persian gum (1:2, 1:1, and 2:1) as the coating wall were produced and dried in a freeze dryer. The optimal properties of emulsions and nanocapsules were achieved at the 10% concentration of extract and the 1:1 ratio of maltodextrin/Persian gum as the wall material. The release rate of phenolic compounds reached its maximum value (88%) after 60 days.

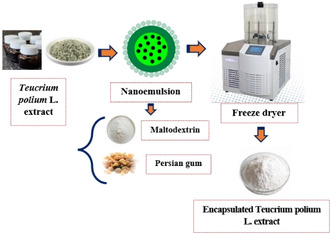

## 1 INTRODUCTION

Medicinal plants have been used therapeutically and nutritionally since ancient times. Several studies have shown that these plants contain abundant bioactive compounds, such as alkaloids, tannins, flavonoids, and phenolic compounds, which have antioxidant and antimicrobial properties. Due to their availability, natural essential oils and herbal extracts are more affordable and widely used today. On the other hand, synthetic antioxidants are very expensive compared to natural antioxidants. Bacterial resistance to antibiotics has also become an issue due to excessive antibiotic use. Therefore, it is essential to find novel antimicrobial compounds with minimal side effects (Kaur et al., 2021).

Felty germander is a medicinal plant that grows in different climates and belongs to the *Labiatae* family. Chemical compounds in this herb include α‐pinene, β‐pinene, verbenone, β‐myrcene, spathulenol, kobnerole, limorone, D‐germacrene, polyphenols, and trans‐caryophyllene, which are soluble in nearly all solvents. The bioactive components in these compounds can exhibit significant antimicrobial, anti‐inflammatory, anti‐diabetic, and antioxidant properties. Numerous studies have highlighted the superior antimicrobial efficacy of felty germander essential oil compared to gentamicin. However, the vulnerability of these extracts and essential oils to adverse conditions, including high temperatures and oxygen exposure, restricts their applicability in food. Furthermore, their effectiveness is hindered by challenges in the release rate. In this regard, several strategies, such as encapsulation, are presented as solutions to these challenges. Encapsulation technology involves encapsulating solids, liquids, or gases in a matrix or capsule. This process involves applying a thin coating to small solid particles, liquid droplets, and gas bubbles to form a capsule that protects the contents from adverse environmental conditions. This capsule protects the contents from oxygen, heat, chemicals, and biological factors (Lipin & Lipin, 2022). Following this, the capsulated components will be released at a controlled and specified rate in response to pH changes, shear stress, temperature, enzymatic activity, and osmotic pressure. The encapsulation methods include spray drying, freeze drying, fluidized‐bed coating, extrusion, and liposome entrapment. There are three main objectives in all these processes: forming a protective wall around the substance, preventing leakage and penetration of the encapsulated substance during storage, and preventing environmental factors from interacting with the core (Nikjoo et al., 2020). Different studies have been conducted on the encapsulation of herbal compounds, such as marjoram extract (Akbarbaglu et al., 2018), cardamom essential oil (Shahidi & Molaveasi, 2020), peppermint extract (Nikjoo et al., 2020), and watermelon juice (Oberoi & Sogi, 2015).

In general, an emulsion is the dispersion of a liquid into another, where a mobile phase is dispersed into the continuous phase as small, spherical droplets. The preparation of a stable emulsion is a critical part of the encapsulation process. The most influential factors in the production of emulsions are droplet size, stability, viscosity, and method of production. Colloidal systems are generally thermodynamically unstable in the vast majority of cases. However, surface‐active compounds (surfactants) can produce kinetically stable emulsions. Consequently, the capsule materials must have high emulsification ability and low viscosity at high concentrations. Additionally, they must be capable of releasing substances readily and efficiently at the appropriate time. However, a challenge encountered by this technique is the restricted range of available materials. The coatings must exhibit emulsifying properties, enable film formation, refrain from reacting with the core substance, be cost‐effective, and allow for swift drying. Polysaccharides are desirable for this purpose due to their low viscosity, solubility, emulsifying ability, and preservation of volatile compounds (Nikjoo et al., 2020).

For this reason, the current study used maltodextrin and Persian gum to encapsulate felty germander extract. Maltodextrin is a mixture of compounds with a molecular weight of less than 20 dextrose equivalents (DE) and a molecular weight between polysaccharides and oligosaccharides. Network formation, high solubility, low viscosity, neutral taste and aroma, and reasonable price have made maltodextrin appropriate for being a wall to protect the compounds sensitive to oxidation. In addition, maltodextrin reduces the adhesion of substances to the dryer walls by raising the glass transition temperature and forming a film around the atomized droplets (Goula & Adamopoulos, 2008).

Persian gum, also known as Zedo or Angum, is a transparent secretory gum sourced from wild almond trees (*Amygdalus scoparia*) subjected to stress factors such as high temperatures, humidity, and insect bites. The gum is categorized into two grades based on color changes. The first grade, termed “ink,” exhibits a white to cream color. In the second grade, two primary colors, yellow and red, are predominant. There are numerous medicinal, industrial, and food applications for Persian gum, including its use as an emulsifier and suspended agent. According to chemical analysis, Persian gum is an anionic polysaccharide of glucose and arabinose units composed predominantly of glucose units and contains two insoluble (70%–75%) and water‐soluble (25%–30%) sections (Mohammadi Golchin et al., 2021). Food encapsulation should be made using food‐grade materials that are safe for human consumption. Microcrystalline cellulose is one of the most common cellulose derivatives. It is a polymer consisting of β‐1,4‐glucose units, naturally formed from wood cellulose. Microcrystalline cellulose is used as a fat substitute in food products. In foods, microcrystalline cellulose solution has a similar flow behavior to oil‐in‐water emulsions and generates a soft, jelly‐like texture. The presence of a hydrophilic compound beside microcrystalline cellulose facilitates its water absorption and the production of all kinds of colloidal microcrystalline cellulose. As part of the encapsulation process, microcrystalline cellulose forms a core, in which the capsule membrane material accumulates on top of each other in layers. Furthermore, due to its porous spherical structure, moist granules dry and clean quickly, adhesion and clumping are reduced, particle flow is increased, and compressibility is more readily achieved (Mohammadi Golchin et al., 2021). Based on our investigations, the availability and selection of the most effective factors relating to emulsion stability have been identified as one of the main challenges for food industry scholars. In reviewing the literature, it has been discovered that no research has been conducted on encapsulating *Teucrium polium* L. extracts, not only in Iran but also worldwide. Thus, this research investigated the production of a stable emulsion of *Teucrium polium* L. extract, maltodextrin, and Persian gum using the response surface method. This emulsion was then applied in a nanoencapsulation process using the freeze‐drying method.

## 2 MATERIALS AND METHODS

### 2.1 Materials

Felty germander was gathered from Aligudarz city in Lorestan province in the early spring of 2022. The plant's upper parts were carefully dried in the shade and stored in polyethylene bags in a freezer at −18°C. All reagents and standards were procured from Sigma‐Aldrich Company, and for optimal quality, maltodextrin, Persian gum, microcrystalline cellulose, and microcrystalline cellulose, along with all of the chemical substances and solvents, were purchased from Merck Company at their highest purity (98%).

### 2.2 Methods

#### 2.2.1 Extraction of *Teucrium polium* L. extract

Prior to the extraction process, the herbal samples underwent pulverization using a grinder (Bosch TSM6A011 model) and were subsequently sieved through a 50‐mesh sieve. The extraction was conducted using a two‐component solvent mixture of water and ethanol (25% water: 75% ethanol), following the Jayaprakasha method with some modifications (Jayaprakasha et al., 2003). For this procedure, 10 g of felty germander plant powder was precisely measured (Perten Co, Sweden, ±0.01 g) and placed into an Erlenmeyer flask containing 100 mL of the designated solvent. The mixture was stirred at 280 rpm for 5 hours in a shaker incubator set at 25°C. Subsequently, the extracted extracts were separated from the herbal solid components using Whatman filter paper No. 1. The obtained extracts were then concentrated using a vacuum rotary evaporator (IKA‐RV05, Germany) at 40°C (to preserve phenolic compounds) and a speed of 200 rpm. Finally, the concentrated extracts were dried and powdered using a freeze dryer (Operun‐FDB550, South Korea) at −50°C. The resulting powders were carefully packaged in polyethylene bags and stored in a freezer at −18°C (Martino et al., 2006).

#### 2.2.2 Preparation of the emulsions

The continuous phase in this study comprised a blend of maltodextrin and Persian gum. The mixture of the wall material (maltodextrin: Persian gum) was dissolved in deionized water in three different ratios (1:2, 1:1, and 2:1) to formulate the emulsion. Additionally, 1 g of Tween 80 was introduced into the mixture as an emulsifier. Subsequently, felty germander extract was incorporated into the solution at three different concentrations (10%, 20%, and 30%). Utilizing an Ultra‐Turrax homogenizer at 25,200 *g* for 10 min at 10°C, a thoroughly homogenized solution and a uniform emulsion were achieved, aiming to reduce the particle size to the nanoscale (Chranioti & Tzia, 2013).

#### 2.2.3 Freeze‐drying of the emulsions

The produced nanoemulsions were subjected to freezing at −20°C for 24 h, followed by a 48‐hour drying process in a freeze dryer under a pressure of 0.017 mPa at −50°C. Subsequently, the extracts retrieved from the freeze dryer underwent powdering using a mortar and crucible and were subsequently stored at a temperature of −20°C (Chranioti & Tzia, 2013).

#### 2.2.4 Emulsions tests

##### Emulsion stability

To assess emulsion stability, 15 mL of the emulsion was dispensed into a McCarty glass and preserved for 6 weeks at 4°C. At the end of each week, the length of the surface, representing the turbid creamy layer, was measured in millimeters and compared to the total length of the emulsion (Chanamai & McClements, 2001).

(1) $$\text{Creaming}=\frac{\;\text{Lenght of cream}\;\left(\mathrm{mm}\right)\;}{\text{Total lenght of emulsion}\;\left(\mathrm{mm}\right)\;}$$

##### Particle size and polydispersity index

The particle size and polydispersity index of the prepared nanoemulsions were assessed using a laser light refraction device (Nano‐Zetasizer, Malvern, England) (Joye et al., 2015).

##### Measuring zeta potential

The measurement of zeta potential was conducted using the Zetasizer device (Nano‐Zetasizer, Malvern, England). The apparatus was equipped with an electrochemical cell housing two electrodes. Initially, the samples were diluted with deionized water at a ratio of 1:5 and subsequently introduced into the cell. The velocity of particle motion was then measured, following the methodology presented by Joye et al. (2015).

##### Apparent viscosity

Immediately after the preparation of samples, the apparent viscosity of emulsion samples was measured by a Brookfield rotational viscometer (RVDV‐ΙΙ^+^, US) (Chanamai & McClements, 2001).

#### 2.2.5 Nanocapsules tests

##### Moisture content

In this research, 2 g of the obtained powder was dried in an oven at 105°C for 24 h until it reached a stable weight. Afterward, the moisture contents of the samples were calculated using equation (2) (Mohammadi Golchin et al., 2021).

(2) $$M\left(\%\right)=\frac{W_{2}-W_{3}}{W_{2}-W_{1}}\times 100$$

In which; M = moisture content (%); $W_{1}$ = weight of the empty container (g); $W_{2}$ = total weight of powder and container (g); and $W_{3}$ = total weight of dried powder and container after placing in oven (g).

##### Encapsulation efficiency

In order to measure the encapsulation efficiency, 200 mg of the capsule was poured into 2 mL ethanol and stirred for 1 min. Then, the obtained mixture was centrifuged for 2 min (1008 *g*). After that, the total phenolic content of the surface solution was measured using the Folin–Ciocalteu method and absorbance at 740 nm by the spectrophotometer (Robert et al., 2010).

(3) $$\text{Encapsulation Efficiency}\;\left(\%\right)=\frac{W_{1}-W_{2}}{W_{2}}\times 100$$

In which, $W_{1}$ = the amount of extract in the surface solution of specified amounts of nanocapsules and $W_{2}$ = the amount of added extract for preparation of the same amount of nanocapsules, expressed as mg gallic acid equivalent.

##### Bulk density

To determine the bulk density, 5 g of the produced powder was poured into a 10‐mL graduated cylinder. Then, according to equation (4), the bulk density was calculated using the powder mass ratio to the occupied volume in the cylinder (Rozbahani et al., 2019).

(4) $$\rho_{B}=\frac{m}{v}$$

In which, $\rho_{B}$ = bulk density (kg/m^3^); *m* = powder mass (kg); and v = volume (m^3^).

##### Antioxidant activity

For the investigation of antioxidant activity, 0.1 g of nanoencapsulated extract powder was dissolved in 10 mL of distilled water and further diluted at a ratio of 1:10. Subsequently, 2 mL of the diluted solution was combined with 2 mL of 0.2 mM methanolic DPPH solution and left in darkness for 30 min at 25°C. Following this incubation, the absorbance of the samples was measured using a spectrophotometer device (UV–visible, Model Shimadzu, UV‐160A, Japan) at 517 nm (Khalafi et al., 2016).

#### 2.2.6 The morphology of capsules

Scanning Electron Microscope device (SEM) (Pemteron PS‐230, South Korea) was utilized to examine the outer and surface morphology of particles.

#### 2.2.7 Measurement of the release rate

The release rate of the extracts was measured at 25°C for 8 weeks and every 15 days according to the Folin–Ciocalteu method. In this regard, 5 g of capsules were mixed with 3 g of phosphate buffer (pH = 7), and then the solution was centrifuged at 2268 *g* and room temperature for 90 min. After collecting the lower phase, the total amount of phenolic compounds was measured (Equation 5) and reported in terms of gallic acid (Kalušević et al., 2017).

(5) $$\mathrm{Rr}\;\left(\%\right)=\frac{\mathrm{RPC}}{\mathrm{TPC}\;}\times 100$$

where, Rr = rate of release (%); RPC = percentage of released phenolic compounds; and TPC = percentage of total phenolic compounds.

#### 2.2.8 Statistical analysis of data

The Response Surface Method (RSM) was applied in the present research to optimize and investigate the effects of different treatments. RSM designs the experiment matrix based on the number of variables and the maximum and minimum ranges determined for each variable. Due to the experiment layout, RSM provides reliable statistical results, even without repetition. In addition to facilitating research, RSM also saves time and reduces side costs. This study employed a central composite design with two variables: the first being the wall material (maltodextrin: Persian gum in three ratios – 1:2, 1:1, and 2:1), and the second being felty germander extract in three concentrations (10%, 20%, and 30%). This design aimed to explore the influences of these factors on the dependent variables (responses). In equation (6), Y is the estimated response, while $\beta_{0}$ represents the constant coefficient, $\beta_{1}$ and $\beta_{2}$ are the linear effects, $\beta_{11}$ and $\beta_{22}$, indicate the quadric effects, and $\beta_{12}$ shows the interaction effect.

(6) $$Y=\beta_{0}+\beta_{1}x_{1}+\beta_{2}x_{2}+\beta_{11}x_{1}^{2}+\beta_{22}x_{2}^{2}+\beta_{12}x_{1}x_{2}$$

## 3 RESULTS AND DISCUSSION

The analysis of estimated regression coefficients in the second‐degree polynomial model for response variables is displayed in Tables 1 and 2.

**TABLE 1:** Estimated regression coefficients in the second‐degree polynomial model for response variables of emulsion.

Source	ES	*F*‐value	*p*‐Value	EDS	*F*‐value	*p*‐value	PDI	*F*‐value	*p*‐value	ZP	*F*‐value	*p*‐value	VIS	*F*‐value	*p*‐value
$\beta_{0}$	−12.08	521.67**	<.0001	+164.346	5.80*	.0567	+1.722	1568.9**	<.0001	+6.598	9.028E+0**	<.0001	+243.41	9.028E+05**	<.0001
$\beta_{1}$	+1.418	710.53**	<.0001	+0.4554	3.92^ns^	.1188	−0.0677	686.43**	<.0001	+0.403	15310.57**	<.0001	+1.7876	15310.57**	<.0001
$\beta_{2}$	−3.32	877.19**	<.0001	−14.196	13.17**	.0222	−1.352	3302.8**	<.0001	−72.218	1.45E+06**	<.0001	+210.97	1.451E+06**	<.0001
$\beta_{11}$	−0.0078	15.25**	.0175	−0.00454	0.0479^ns^	.8374	+0.0011	279.37**	<.0001	+0.007	748.70**	<.0001	+0.1418	748.70**	<.0001
$\beta_{22}$	+3.72	346.83**	<.0001	+4.956	5.71*	.0752	+0.332	2249.4**	<.0001	+25.72	9.74E+05**	<.0001	−71.651	9.749E+05**	<.0001
$\beta_{12}$	−0.328	58.98**	.0015	+0.0366	0.0068^ns^	.9382	+0.018	147.88**	.0003	−0.2082	1396.50**	<.0001	−1.0176	1396.50**	<.0001
Lack of Fit	–	1.06^ns^	.2145^ns^	–	2.31^ns^	.11^ns^	–	3.05^ns^	.235^ns^	–	2.77^ns^	.389^ns^	–	2.77^ns^	.389^ns^
*R* ^*2*^	.98	–	–	.97	–	–	.98	–	–	.96	–	–	.98	–	–

*Note*: $\beta_{1}$ = Extraction, and $\beta_{2}$ = Maltodextrin/Gum, *Significant at .05 level, **Significant at .01 level, *p*‐values less than .0500 indicate model terms are significant.

Abbreviations: EDS, Emulsion droplet size; ES, Emulsion stability; ns, Not significant; PDI, Poly disparity index; VIS, Viscosity; ZP, Zeta potential.

**TABLE 2:** Estimated regression coefficients in the second‐degree polynomial model for response variables of nanocapsule.

Source	MC	*F*‐value	*p*‐value	EE	*F*‐value	*p*‐value	PD	*F*‐value	*p*‐value	AA	*F*‐value	*p*‐value
$\beta_{0}$	−3.74600	1646.05	<.0001	+82.72800	17111.10**	<.0001	−0.318000	16.40**	0.0090	+45.74800	18586.92	<.0001
$\beta_{1}$	+0.347600	4215.05	<.0001	+0.500700	11048.44**	<.0001	+0.027300	46.91**	0.0024	+1.20070	29534.04	<.0001
$\beta_{2}$	+1.77100	867.15	<.0001	+13.63700	32809.71**	<.0001	+0.193000	0.9574^ns^	0.3832	−0.573000	2659.90	<.0001
$\beta_{11}$	−0.005860	527.49	<.0001	−0.017370	2745.38**	<.0001	−0.000230	1.61^ns^	0.2736	−0.023670	5097.99	<.0001
$\beta_{22}$	−0.351000	189.25	<.0001	−4.42700	17832.87**	<.0001	−0.033000	3.31*	0.1430	−0.787000	563.58	<.0001
$\beta_{12}$	+0.013400	6.03	<.0001	−0.050200	50.16**	<.0001	−0.003800	0.9601^ns^	0.3826	+0.113800	257.77	<.0001
Lack of Fit	–	1.44^ns^	.140^ns^	–	3.06^ns^	.089^ns^	–	2.08^ns^	0.089^ns^	–	1.18^ns^	.156^ns^
*R* ^2^	.96	–	–	.98	–	–	.98	–	–	.97	–	–

*Note*: $\beta_{1}$ = Extraction, and $\beta_{2}$ = Maltodextrin/Gum, *Significant at .05 level, **Significant at .01 level, *p*‐values less than .0500 indicate model terms are significant.

Abbreviations: AA, Antioxidant activity; EE, Encapsulation efficiency; MC, Moisture content; ns, Not significant; PD, Powder density.

### 3.1 Evaluation of the emulsion properties

#### 3.1.1 Emulsion stability

According to Table 1, the effect of extract concentration ($\beta_{1}$), different ratios of maltodextrin and gum ($\beta_{2}$), the effect of the second‐order extract concentration ($\beta_{11}$), the effect of the second‐order of different ratios of maltodextrin and gum ($\beta_{22}$), and the interaction effect of extract concentration and different ratios of maltodextrin and gum ($\beta_{12}$) on the emulsion stability are significant (p≤.01). Furthermore, the explanatory coefficient (*R* ^2^), *F*‐value, and *p*‐value demonstrate that the proposed model fits well.

**FIGURE 1:**
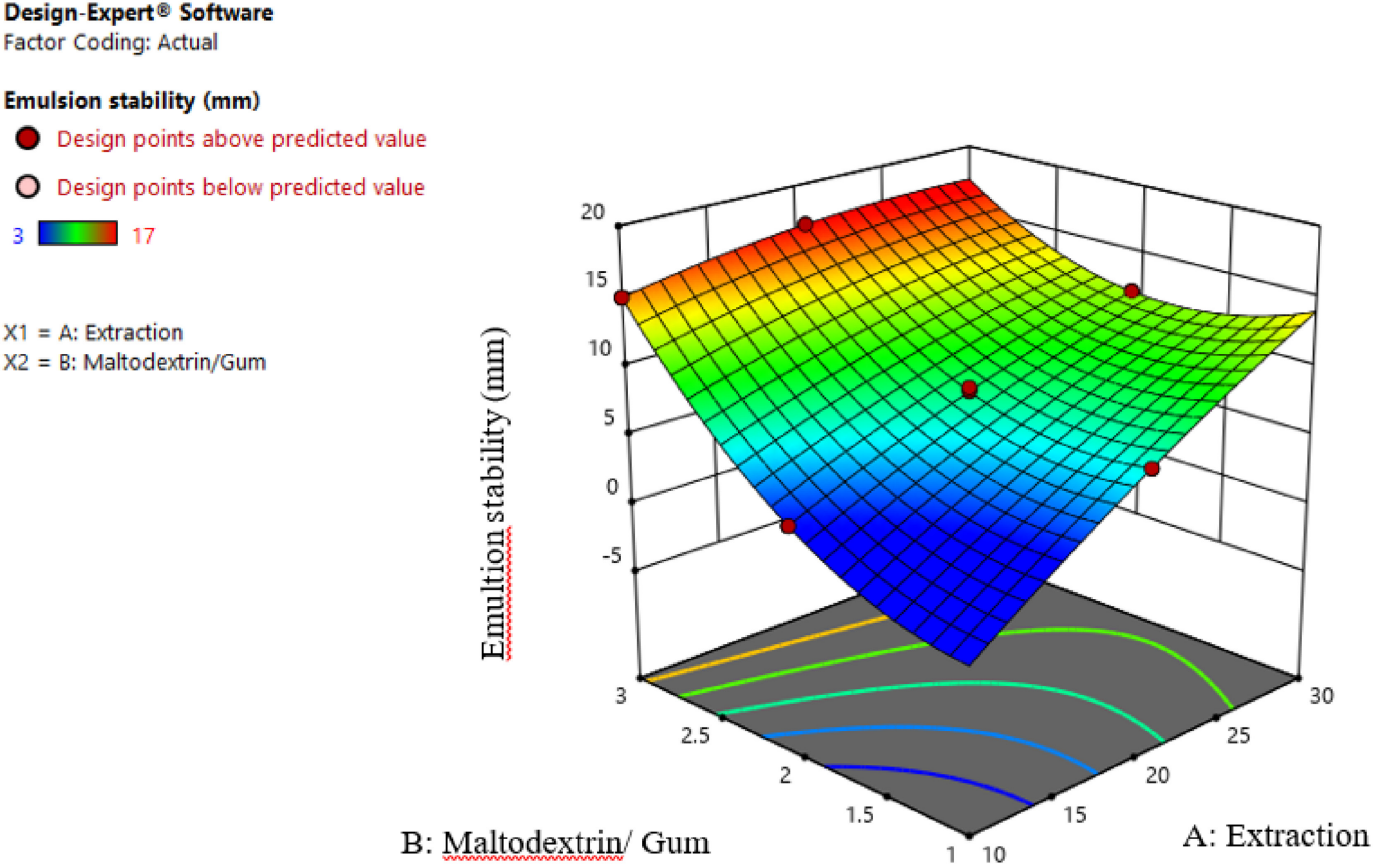
Interaction effect of extract concentration and different ratios of maltodextrin and gum on the emulsion stability.

Creaming is a sign of instability, causing emulsions to separate into two phases. Emulsion properties, such as stability, viscosity, droplet size, free oil content, and oxidative stability, are affected by the type and mixture of materials used for microencapsulation walls. It is reported that certain features of gums, such as hydrophilicity/hydrophobicity index, acidic/alkaline index, and the presence of particular chemical groups, can affect the absorption and emulsification of gums in the emulsion (Carneiro et al., 2013). In the present study, Persian gum‐containing emulsions showed more stability (Figure 1). In other words, increasing the maltodextrin ratio to Persian gum decreased the stability of the emulsion. These changes can be investigated in relation to the size of the droplets and how they are distributed in the dispersed phase. Due to the fact that emulsion stability depends on a variety of factors, including droplet size, this result is probably an indication that the larger droplets contain a higher ratio of maltodextrin, which results in the formation of two phases. On the other hand, the possibility of colliding and adhesion and creating larger droplets would be increased by a higher motion of particles, leading to creaming. This result can be analyzed using Stockes' law. According to this law, the velocity of particle sediment in a fluid is directly related to gravity force, the discrepancy between particle and fluid density, and the square of particle radius. Moreover, the sedimentation rate is inversely related to fluid viscosity (Yuan et al., 2008). Thus, the reduction in droplet size increases the emulsion's stability. Since increasing the amount of maltodextrin increased the size of droplets and the velocity of particle movement in the emulsion system, emulsions containing higher ratios of Persian gum to maltodextrin were more stable. Additionally, increasing the extract concentration decreased emulsion stability, probably due to larger droplets (Esmaeili & Saremnia, 2012).

#### 3.1.2 Droplet size

According to Table 1, the effect of different ratios of maltodextrin and gum ($\beta_{2}$) and the effect of the second‐order extract of different ratios of maltodextrin and gum ($\beta_{22}$) on the sample's droplet size are significant (p≤.01). Also, the explanatory coefficient (*R* ^2^), *F*‐value, and *p*‐value represent a desirable fit of the proposed model.

**FIGURE 2:**
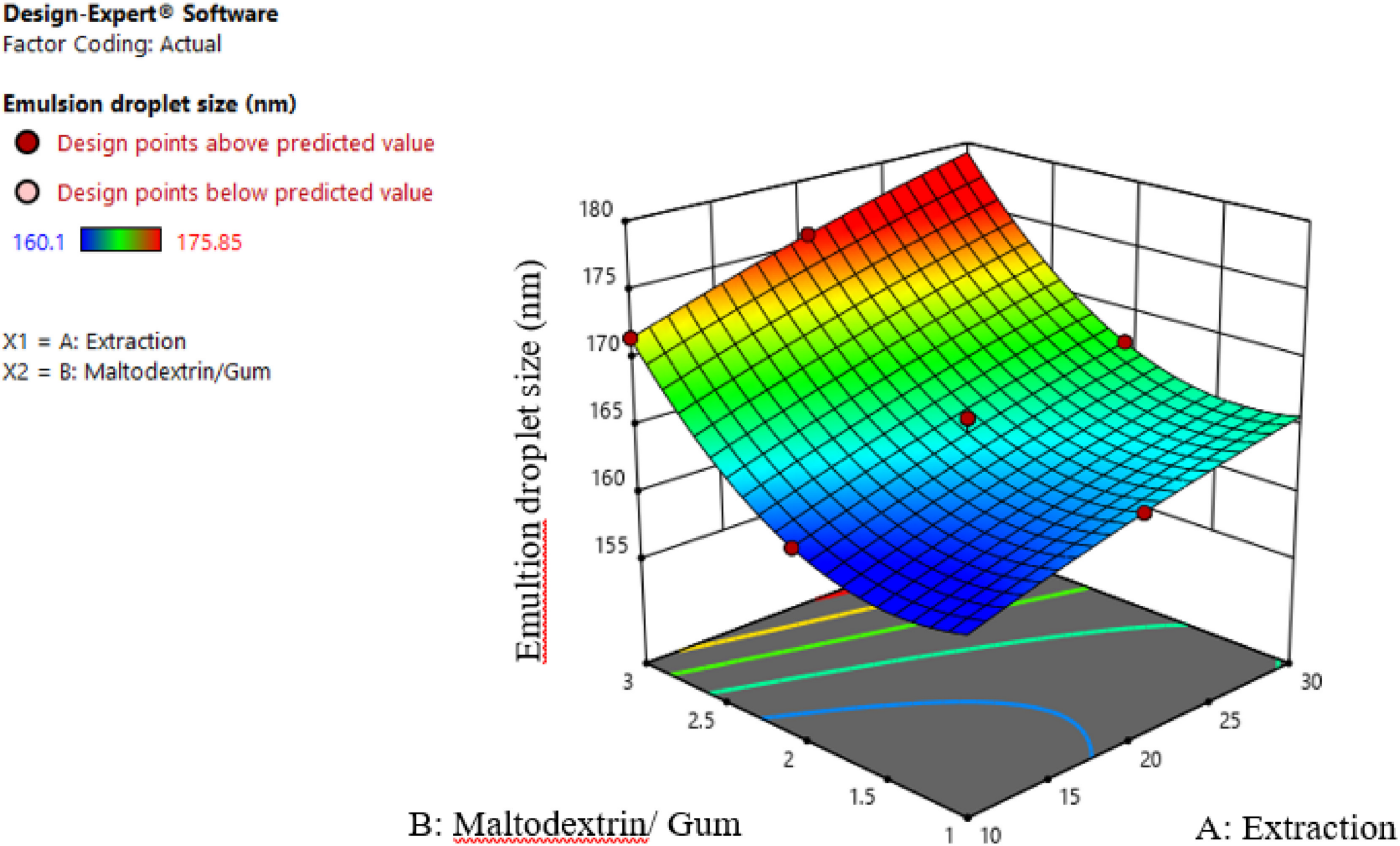
Interaction effect of extract concentration and different ratios of maltodextrin and gum on the droplet size.

Various elements impact the droplet size of an emulsion; the type and amount of used emulsifier, types of phases, emulsion preparation methods, environmental conditions such as pH, and the presence of metal ions are among the critical ones. The capsule materials, molecular bonds, and structure of the applied polymer also affect the size of microencapsulated substances. Typically, the enhanced stability observed in emulsions with smaller droplets can be attributed to increased resistance against gravity due to heightened Brownian motion. This phenomenon not only fosters greater stability but also imparts improved functional properties (Pushpamalar et al., 2016). According to Figure 2, the particles of nanoemulsions produced with a 1:1 ratio of maltodextrin and Persian gum had the smallest size, possibly due to the higher viscosity and slower Brownian motion of the droplets. Moreover, increasing the maltodextrin ratio increased the droplet size. Maltodextrin has no emulsion properties; therefore, its application with an active polymer is favorable (Bule et al., 2010). Similar behavior in the interaction of hydroxymethyl cellulose and sodium dodecyl sulfate was observed by Petrovic et al. (2010). According to Tonon et al. (2011), there is an indirect relationship between particle size and emulsion viscosity, consistent with the present results. Since the Persian gum can reduce the surface tension of water, and the soluble part of the gum solution has more protein content, these compounds can be significantly practical for stabilizing the emulsions. As previously stated, including protein in the gum structure imparts emulsifying properties. Specifically, the protein component plays a crucial role in stabilizing the emulsion by diminishing the size of the droplets (Pushpamalar et al., 2016). Furthermore, in accordance with conducted studies, a notable increase in particle size was observed with escalating extract concentration (Esmaeili & Saremnia, 2012).

#### 3.1.3 Polydispersity index (PDI)

According to Table 1, the effect of extract concentration ($\beta_{1}$), different ratios of maltodextrin and gum ($\beta_{2}$), the effect of second‐order extract concentration ($\beta_{11}$), the effect of second‐order of different concentrations of maltodextrin and gum ($\beta_{22}$), and the interaction effect of extract concentration and different ratios of maltodextrin and gum ($\beta_{12}$) on samples polydispersity index are significant (p≤.01). Moreover, the explanatory coefficient (*R* ^2^), *F*‐value, and *p*‐value represent a desirable fit of the proposed model.

**FIGURE 3:**
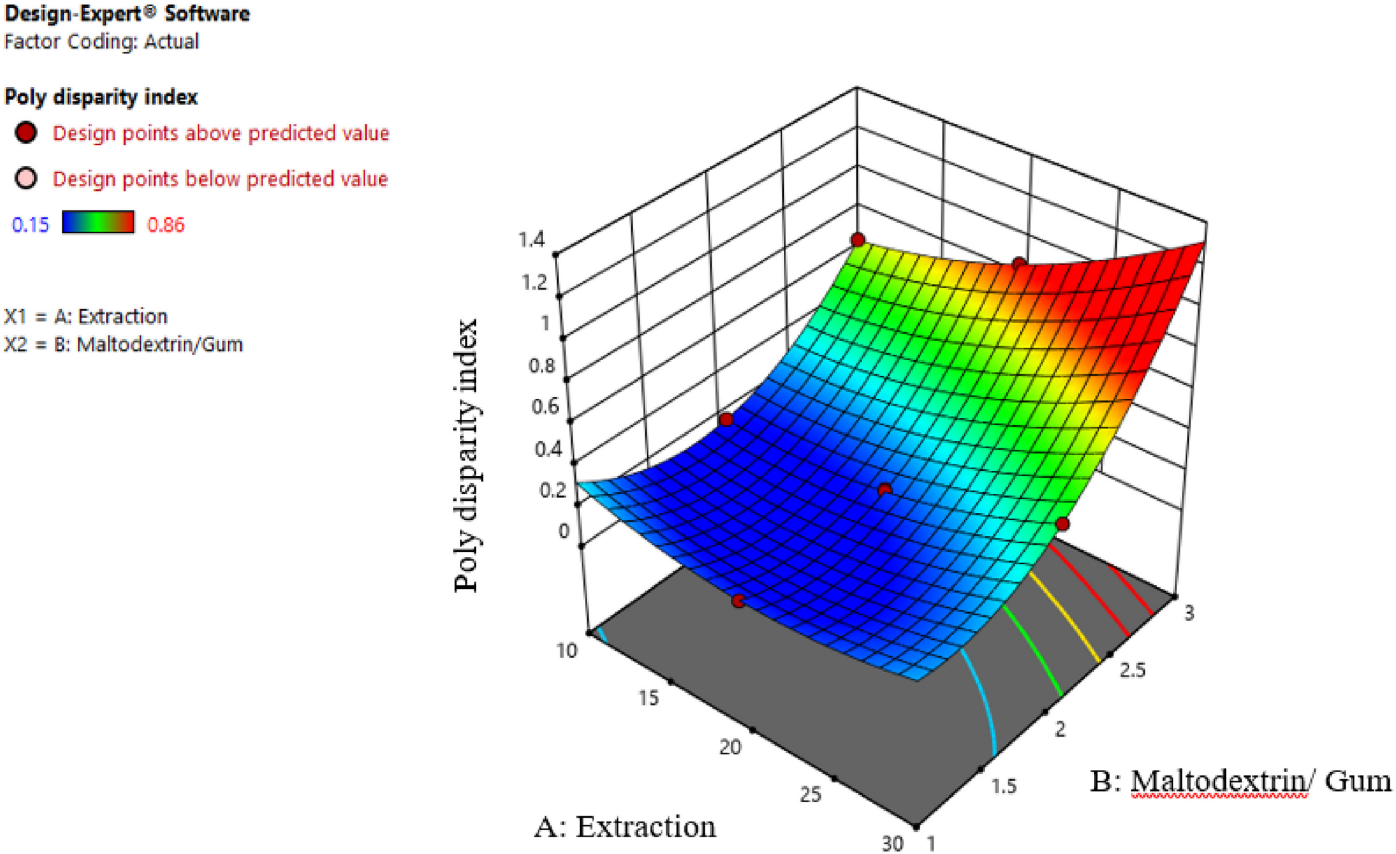
Interaction effect of extract concentration and different ratios of maltodextrin and gum on the polydisparity index.

The polydispersity index (PDI) stands out as a crucial factor influencing the physicochemical properties of nanoemulsions, including their rheological behavior. This, in turn, plays a pivotal role in determining the overall stability of these systems. Lower PDI indicates better dispersion of particles and, subsequently, a more homogenized colloidal system. In general, reduction of particle size and appropriate dispersity can effectively improve properties like viscosity, particle stability, color, nanoemulsion appearance, and creaming. Theoretically, this index is in the range of 0–1, and values higher than 0.5 indicate immense dispersity of particles (Tamjidi et al., 2013). In this research, the value of this index was less than 0.2 for some treatments showing a uniform PDI and, as a result, successful production of nanoemulsion. Based on Figure 3, PDI increased by increasing the particle size. However, by reducing particle size, the index decreased as well. Also, increasing the extract concentration increased PDI due to the increase in the particle size. In this regard, it can be stated that the smaller size of particles decreases gravity impact. Therefore, the flocculation and adhesion possibilities would be significantly reduced (Yuan et al., 2008).

#### 3.1.4 Zeta potential

According to Table 1, the effect of extract concentration ($\beta_{1}$), different ratios of maltodextrin and gum ($\beta_{2}$), the effect of second‐order extract concentration ($\beta_{11}$), the effect of second‐order of different ratios of maltodextrin and gum ($\beta_{22}$), and the interaction effect of extract concentration and different ratios of maltodextrin and gum ($\beta_{12}$) on the zeta potential are significant (p≤.01).

**FIGURE 4:**
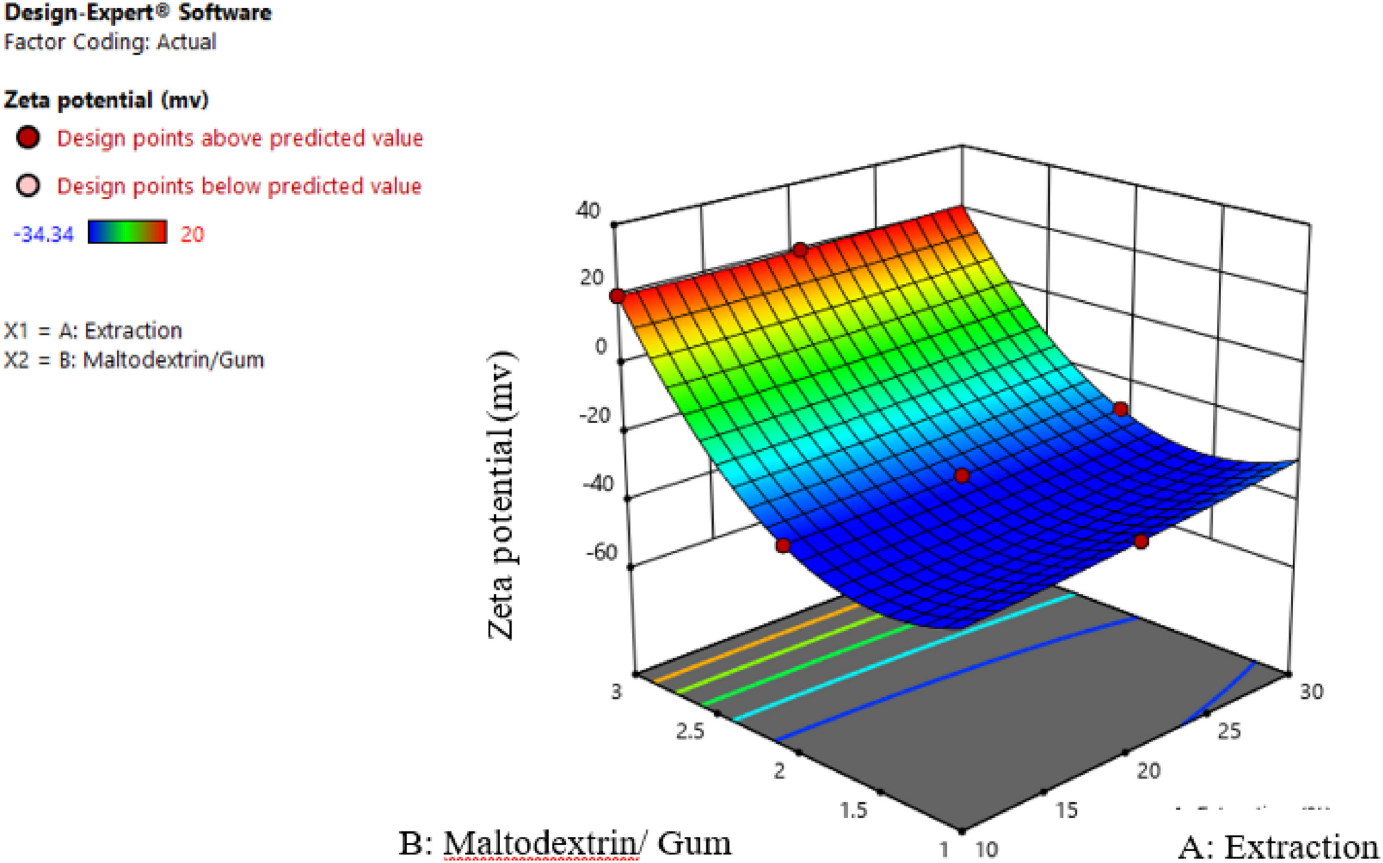
Interaction effect of extract concentration and different ratios of maltodextrin and gum on the zeta potential.

The high zeta potential of emulsion particles raises the electrostatic repulsion force and, subsequently, improves physical stability. The zeta potential value is related to the stability of the colloidal dispersion. Low zeta potential indicates high intermolecular attraction, leading to aggregation, coagulation, and instability. Therefore, the surface charge properties and physical stability of nanoemulsions can be investigated by measuring the zeta potential. Surface charge, electrophoretic mobility, and zeta potential are affected by numerous factors, such as ionic strength, type and concentration of polysaccharide macromolecules and their ratios, and pH. The optimum pH for each emulsion improves its stability due to the increase in zeta potential (Nikjoo et al., 2020). According to Figure 4, the initial stability of the substance was maintained using high concentrations of Persian gum. The soluble component of gum, as a strengthening substance, had a uniform status, and no sediment or colloidal state occurred. In addition to electrostatic repulsion, the spatial obstruction made by gum causes the system to stabilize, avoiding the aggregation of particles. Gum is an acidic polysaccharide; its carboxyl groups establish the anionic basis. As shown, negative charges can further maintain inner contents compared to positive ones. Electrostatically stabilized nanoemulsions require at least 30 mV zeta potential. In this research, the zeta potential was negative for samples containing gum, while it was more than 30 mV in high concentrations. Guzey et al. (2014) stated that an emulsion would be appropriately stable when the zeta potential of its particles was higher than 30 mV. Strong electrostatic repulsion explains this by preventing particles from colliding and, as a result, reducing the possibility of flocculation in the emulsion. The anionic structure of gum can be responsible for the negative zeta potential. The zeta potential of maltodextrin acts as a weak polyelectrolyte compared to gum due to the types of polysaccharide chain stabilization substituents. On the other hand, increasing the extract concentration decreased the zeta potential of samples, seemingly due to the reduction of surface charge.

#### 3.1.5 Apparent viscosity

As Table 1 shows, the effect of extract concentration ($\beta_{1}$), different ratios of maltodextrin and gum ($\beta_{2}$), the effect of second‐order extract concentration ($\beta_{11}$), the effect of second‐order of different ratios of maltodextrin and gum ($\beta_{22}$), and the interaction effect of extract concentration and different ratios of maltodextrin and gum ($\beta_{12}$) on the viscosity of samples are significant (p≤.01). Also, the explanatory coefficient (*R* ^2^), *F*‐value, and *p*‐value represent a good fit for the proposed model.

**FIGURE 5:**
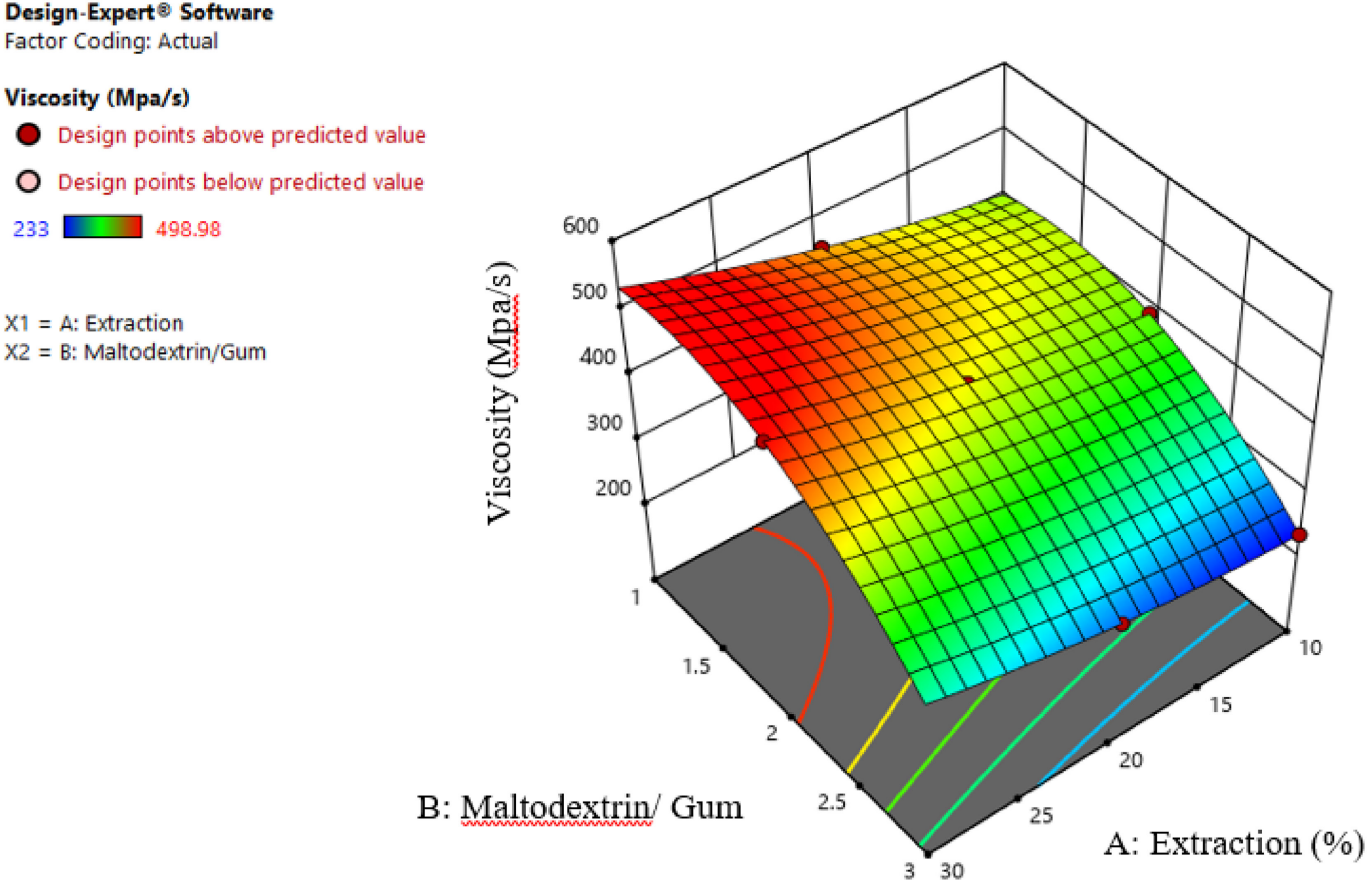
Interaction effect of extract concentration and different ratios of maltodextrin and gum on the viscosity.

Emulsion systems vary in viscosity depending on the type and concentration of wall materials as well as the size of the droplets. The colloidal nature of the continuous phase, the mean droplet size, and the mean dispersity index effectively alter the viscosity (Yuan et al., 2008). Therefore, any alteration in the concentration of polymeric compositions changes the rheological properties of the system (Carneiro et al., 2013). According to Figure 5, increasing the Persian gum raised the viscosity because of the high molecular weight of the gum. In dilute hydrocolloid solutions, molecules exhibit unrestricted and independent movement without collisions. Conversely, higher concentrations increase molecules per unit volume, resulting in aggregation, collisions, involvement, and overlapping. Consequently, the viscosity of the solution rises (García‐Moreno et al., 2016). Generally, emulsion viscosity depends on the molecular weight of the soluble substance, solvent type, and polymer concentration (Weiss et al., 2012). Numerous studies reported increments in the emulsion viscosity because of the molecular weight of the substances. On the other hand, increasing the maltodextrin concentration reduced the viscosity. Other scholars have published similar results (García‐Moreno et al., 2016). To explain this result, the hydrophilicity and hydrodynamic radius will be increased by binding polysaccharides to proteins and unfolding their structure. Eventually, it leads to a lower hydration degree and viscosity. However, it seems that this mechanism does not apply to carbohydrates, whose nature is influential on viscosity (Weiss et al., 2012). The findings of this study suggest that the spatial arrangement of maltodextrin chains on the surface of droplets or the electrostatic‐spatial repulsion between droplets plays a crucial role. This mechanism prevents the close approach of droplets and hinders the entanglement of polysaccharide chains, emerging as a primary factor in creating viscosity. As stated earlier, increasing the viscosity reduced the creaming index. The instability observed in low‐viscosity emulsions may be attributed to the unrestricted movement of oil droplets within the system. Insufficient viscous forces in the aqueous phase cannot effectively hinder the collision of oil droplets, leading to their aggregation. Over time, due to increasing collisions of droplet–droplet, flocculent–droplet, flocculent–flocculent, and Brownian motions, the number of flocculated droplets would increase (García‐Moreno et al., 2016). Petrovic et al. (2010) observed similar behavior in the interaction of hydroxymethyl cellulose and sodium dodecyl sulfate.

### 3.2 Evaluation of nanocapsule properties

#### 3.2.1 Moisture content

According to Table 2, the effect of extract concentration ($\beta_{1}$), different ratios of maltodextrin and gum ($\beta_{2}$), the effect of second‐order extract concentration ($\beta_{11}$), the effect of second‐order of different ratios of maltodextrin and gum ($\beta_{22}$), and the interaction effect of extract concentration and different ratios of maltodextrin and gum ($\beta_{12}$) on the moisture content of powders are significant (p≤.01). Also, the explanatory coefficient (*R* ^2^), *F*‐value, and *p*‐value fit the proposed model well.

**FIGURE 6:**
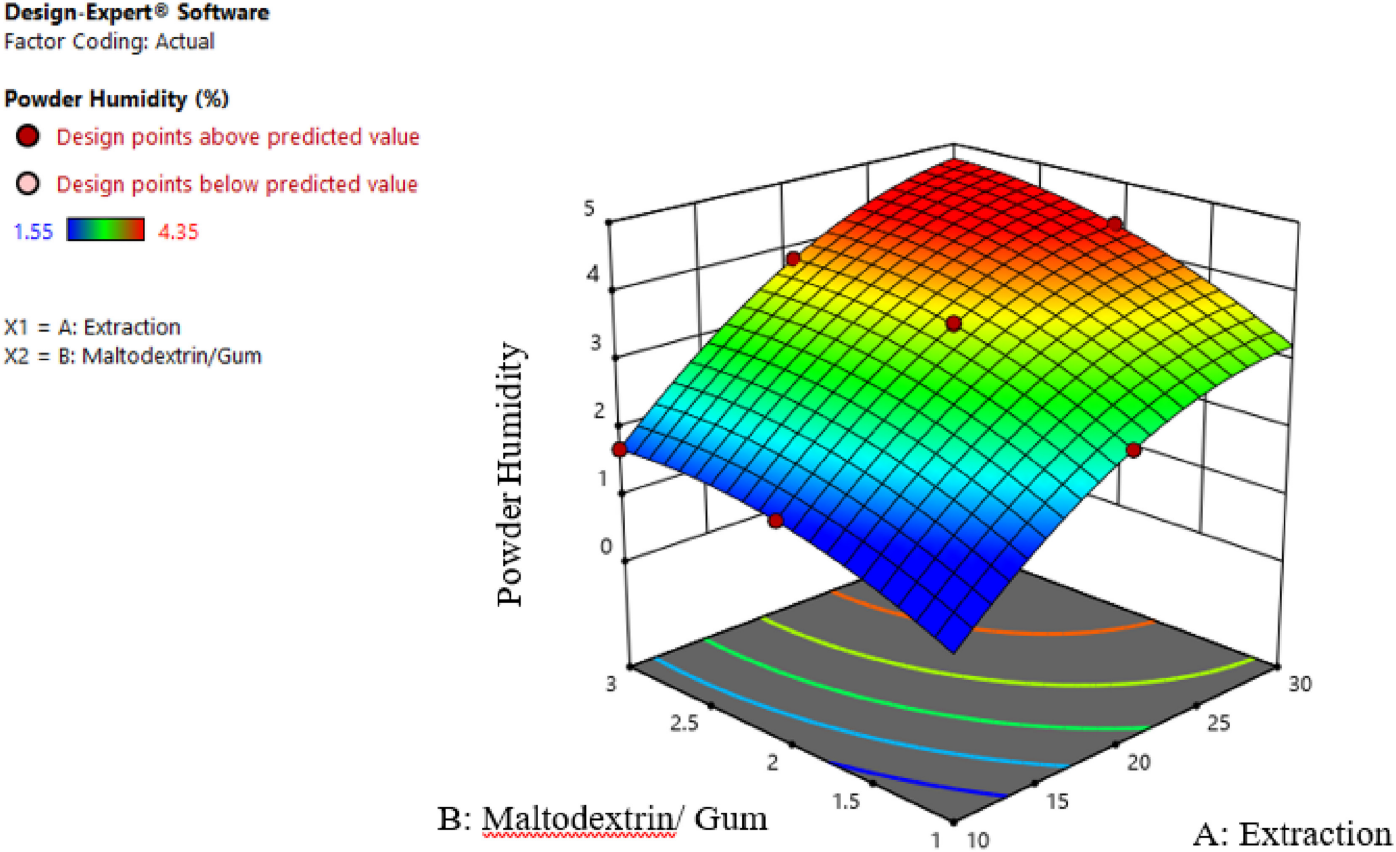
Interaction effect of extract concentration and different ratios of maltodextrin and gum on the moisture content of the samples.

Maintaining a powder humidity level below 4%–5% is crucial, as lower moisture content significantly reduces oxidative decomposition. This, in turn, extends shelf life and enhances consumer acceptability. Less moisture causes less adhesion to powder particles (Koc et al., 2010). The remaining water in the powder affects many technological properties, such as bulk density, solubility, and wettability. Since maltodextrin is a hydrophile non‐ionic compound and Persian gum is a compound with a dual hydrophilic and hydrophobic structure, it seems that by combining these two, a novel wall mixture with appropriate efficiency for nanoencapsulating the extract can be achieved. According to Figure 6, increasing the maltodextrin concentration increased the sample moisture. This phenomenon could be related to the tendency of maltodextrin‐forming sugars to absorb moisture and reduce the free moisture available to evaporate in powders. Also, the presence of large molecules of maltodextrin is an obstacle to the convenient diffusion of water molecules (Sarabandi & Peighambardoust, 2015). The rise in Persian gum content correlates with an increase in the moisture level of powder samples. Persian gum, characterized by its heteropolysaccharide complex with a branched structure, contains hydrophilic groups that attract and bind to water molecules. On the other hand, by increasing the extract concentration, the moisture of powders increased, probably due to reducing the solid substance content of the coating and, consequently, increasing the free moisture available for evaporation (Nikjoo et al., 2020).

#### 3.2.2 Evaluation of encapsulation efficiency

According to Table 2, the effect of extract concentration ($\beta_{1}$), different ratios of maltodextrin and gum ($\beta_{2}$), the effect of second‐order extract concentration ($\beta_{11}$), the effect of second‐order of different ratios of maltodextrin and gum ($\beta_{22}$), and the interaction effect of extract concentration and different ratios of maltodextrin and gum ($\beta_{12}$) on the encapsulation efficiency of the samples are significant (*p* ≤ .01). Also, the explanatory coefficient (*R* ^2^), *F*‐value, and *p*‐value represent the desirable fit of the proposed model.

**FIGURE 7:**
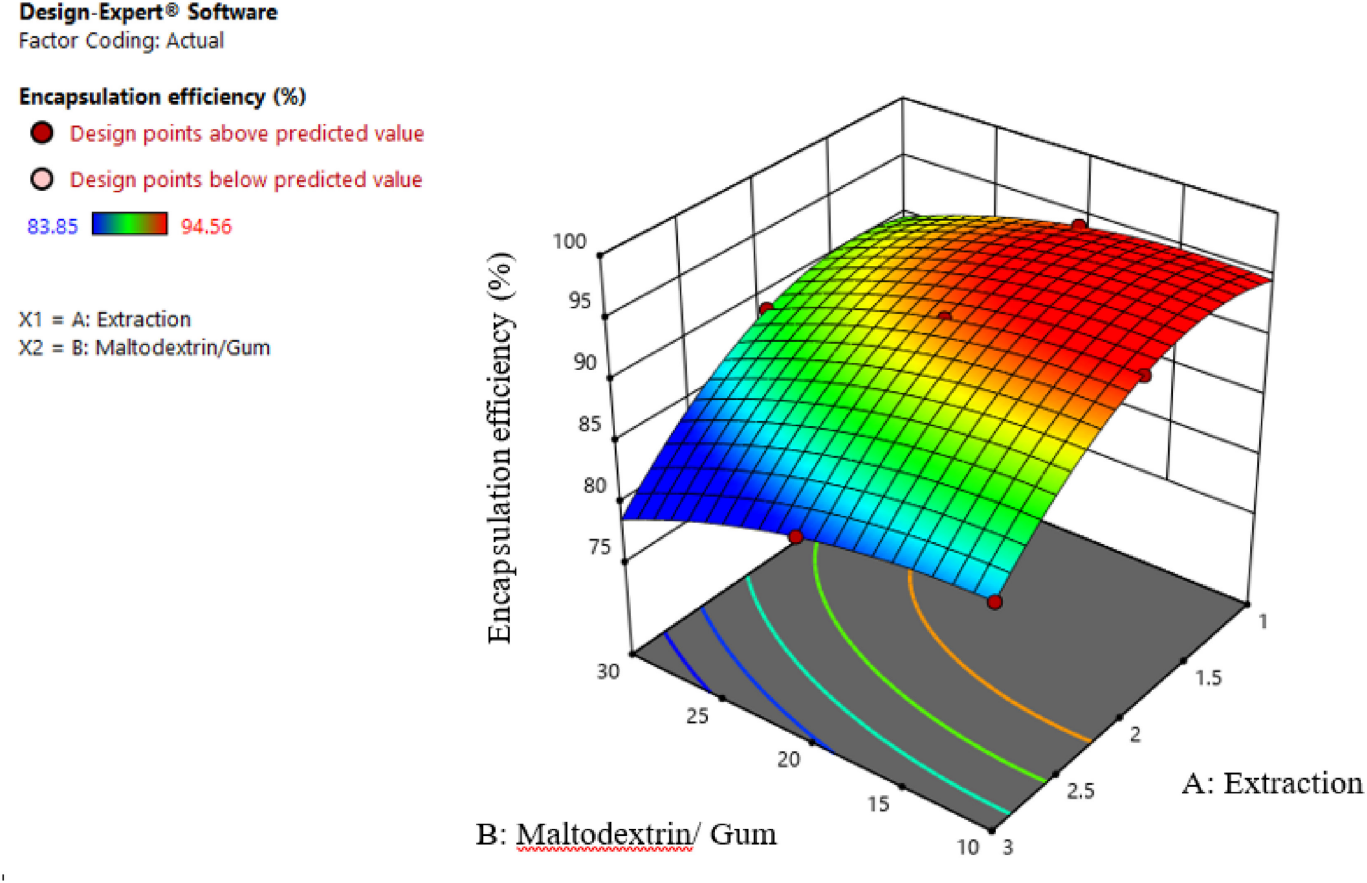
Interaction effect of extract concentration and different ratios of maltodextrin and gum on the microencapsulation efficiency.

The efficiency of the nanoencapsulation process depends on the type and composition of wall materials, the ratio of wall materials to the core, stability, and physicochemical properties of emulsions. Generally, molecular dimensions are crucial in losing phenolic compounds since they are directly related to molecular diffusion. Nanoencapsulated compounds exhibit enhanced stability in emulsions characterized by smaller particle sizes. Consequently, the evaporation of core contents is more efficient in emulsions with smaller particles compared to larger ones (Fernandes et al., 2014). As Figure 7 shows, with an increase in the Persian gum concentration, the efficiency of nanoemulsions improved, probably due to higher stability and viscosity, and as a result, the Brownian motion of the droplets was slowed. The result of the present study is in line with Tonon et al. (2011) results, which reported an inverse relation between particle size and emulsion viscosity. The stabilizing effect of Persian gum in emulsions is attributed to its notable ability to lower the surface tension of water. Additionally, the richness of its soluble part, particularly in terms of protein content, plays a crucial role in enhancing the stability of these compounds in emulsions. Moreover, as mentioned earlier, the emulsifier properties of gum are due to the presence of protein in their structure. This protein part reduces the particle size and increases the stability of the emulsion (Pushpamalar et al., 2016). Various studies have shown that particle size reduction leads to higher microencapsulation efficiency, emulsion stability, and further maintenance of active compounds (Nikjoo et al., 2020; Sarabandi & Peighambardoust, 2015).

#### 3.2.3 Evaluation of bulk density

As exhibited in Table 2, the effect of extract concentration ($\beta_{1}$) and the effect of second‐order extract of different ratios of maltodextrin and gum ($\beta_{22}$) is significant on the bulk density of samples (p≤.01).

**FIGURE 8:**
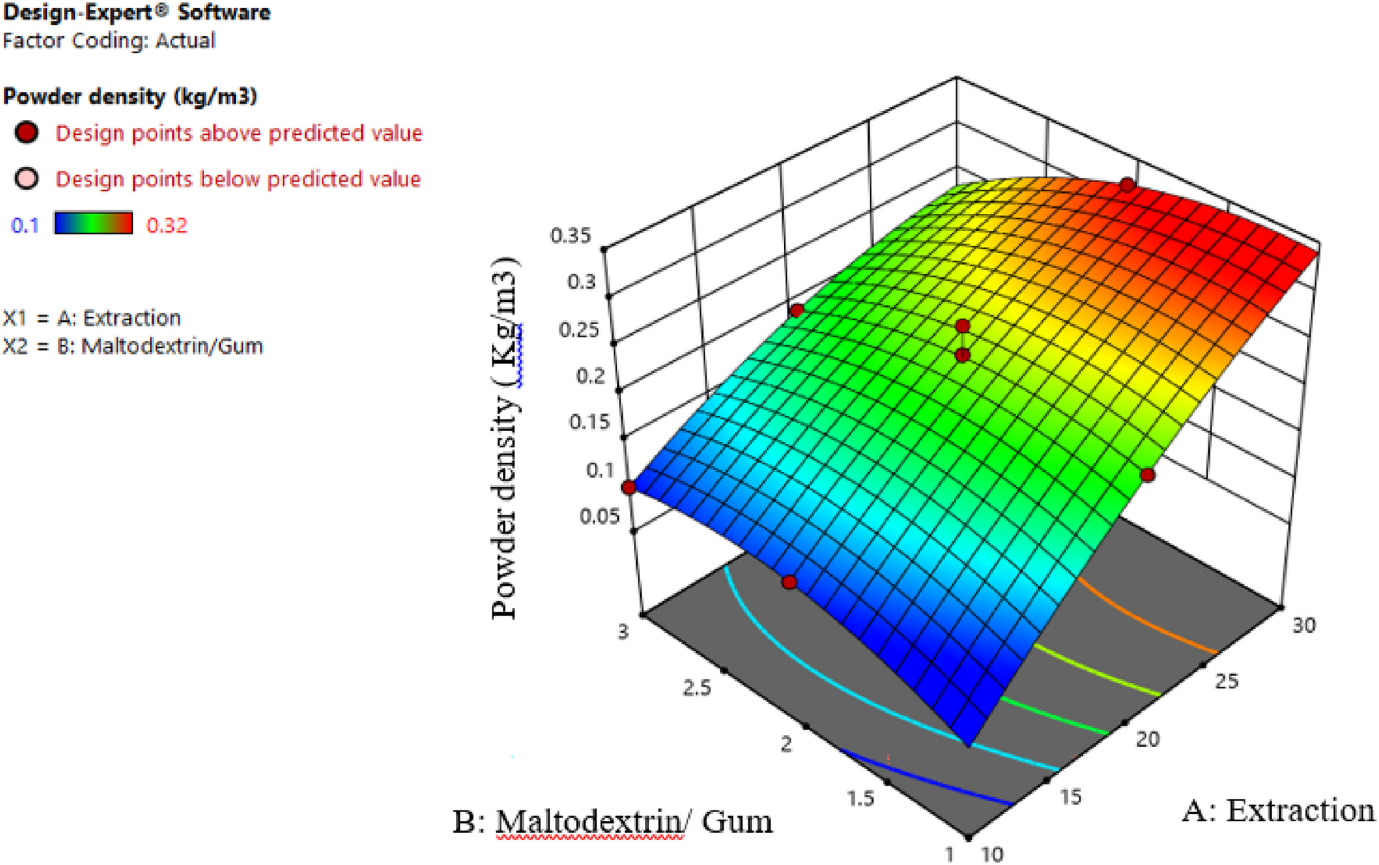
Interaction effect of extract concentration and different ratios of maltodextrin and gum on the bulk density.

Bulk density depends on the size, shape, moisture, chemical composition, and trapped air of particles. These factors depend on feed features, inlet air amount, drying temperature and duration, processing, and transport. The results express that the bulk density increased by increasing the maltodextrin concentration since the bulk mass was higher due to moisture (Sarabandi & Peighambardoust, 2015). Conversely, as moisture levels increase, there is a heightened tendency for particles to adhere to one another. Consequently, the spacing between particles decreases, leading to more powder occupying a specified volume of space. This phenomenon can account for the observed increase in bulk density (Sarabandi & Peighambardoust, 2015). Moreover, by increasing the extract concentration, the bulk density increased. The main reasons for these results include increased particle adhesion, decreased film formation, reduced porosity, and the diminished presence of trapped air within the structure (Nikjoo et al., 2020). As shown in Figure 8, with increasing the Persian gum and sample thickening, the amount of trapped air and, as a result, density increased. Higher moisture content contributes to greater bulk mass due to the presence of water, resulting in denser characteristics than dried samples. Powders containing higher proportions of gum exhibit a higher bulk density than those containing maltodextrin. This can be attributed to the smaller size of gum molecules, which, in turn, produces smaller particles with this carrier, reducing the overall powder volume and justifying the observed increase in density in these treatments.

#### 3.2.4 Evaluating antioxidant activity

According to Table 2, the effect of extract concentration ($\beta_{1}$), different ratios of maltodextrin and gum ($\beta_{2}$), the effect of second‐order extract concentration ($\beta_{11}$), the effect of second‐order extract of different ratios of maltodextrin and gum ($\beta_{22}$), and the interaction effect of extract concentration and various proportions of maltodextrin and gum ($\beta_{12}$) on the antioxidant activity of samples are significant (p≤.01).

**FIGURE 9:**
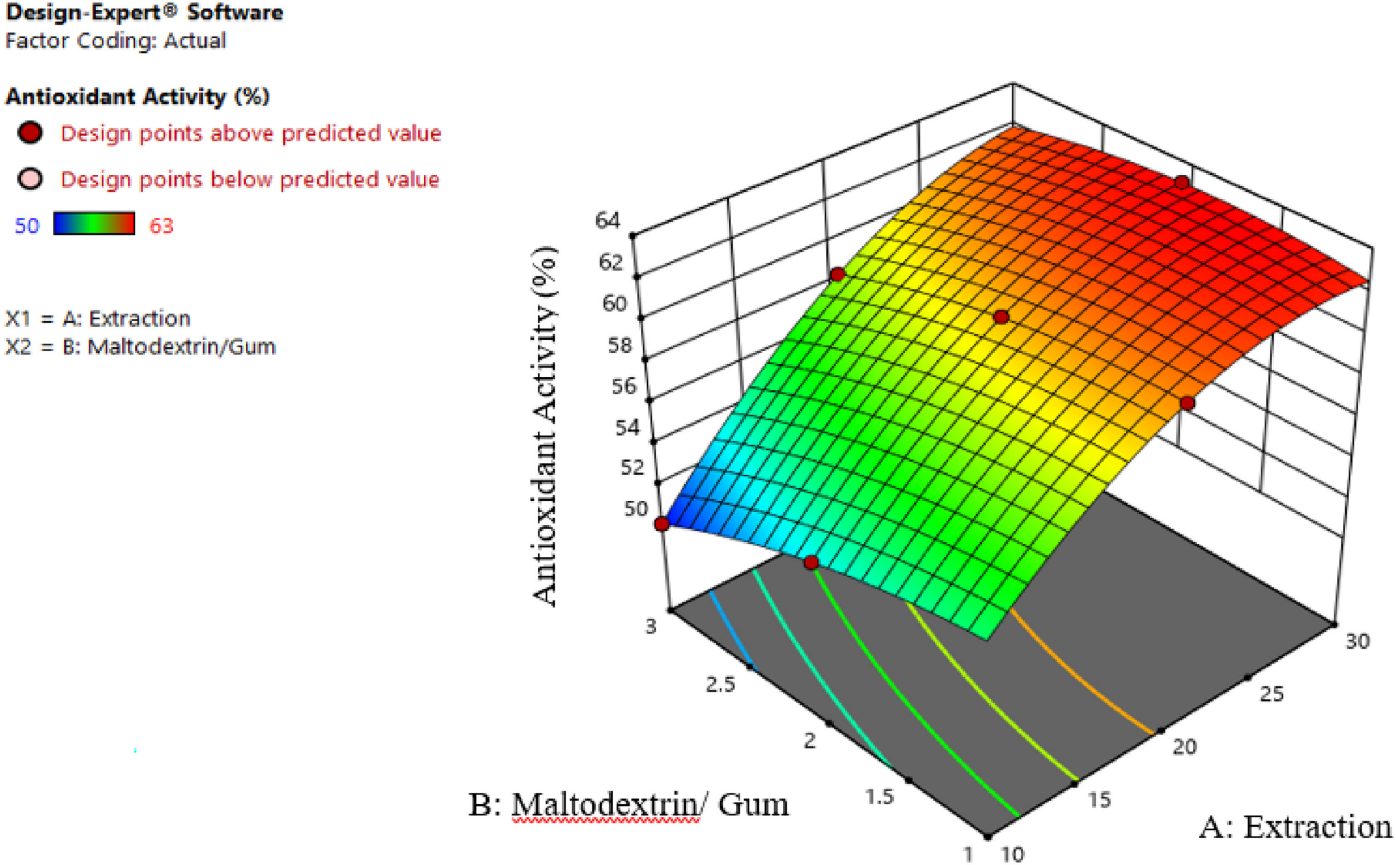
Interaction effect of extract concentration and different ratios of maltodextrin and gum on the antioxidant activity.

According to Figure 9, the antioxidant activity in all studied ratios increased significantly with increasing extract concentration. Also, increasing the maltodextrin and gum ratios (in high ratios) improved the reduction of free radicals since it preserved the phenolic compounds of the extract against environmental degradation (Shahidi & Molaveasi, 2020). Esmaeilzadeh Kenari et al. (2014) stated that the antioxidant activity improved by increasing the extract concentration due to a quantitative increment in phenolic compounds and flavonoids in the extract. However, the antioxidant activity of the nanoencapsulated extract is slightly weaker than that of the free extract. It occurs due to the possible destruction of some phenolic compounds during the drying process (Shahidi & Molaveasi, 2020).

### 3.3 Evaluation of capsule morphology

**FIGURE 10:**
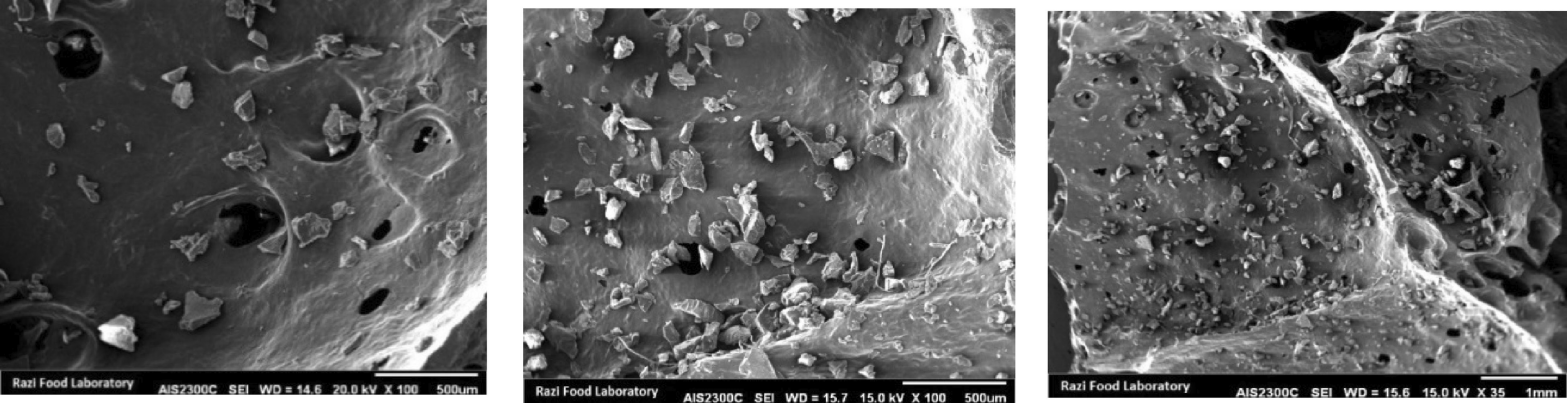
SEM images of nanocapsules in optimal condition.

The effect of the optimum extract, maltodextrin, and Persian gum concentrations on the surface structure and morphological properties of powder containing felty germander extract produced by the freeze‐drying method is displayed in Figure 10. As observed, the produced capsules have no specific geometric shape. The obtained powders are fractured and jagged and show some wrinkles on their surface. Furthermore, multiple cracks and fissures can be seen on the particles. This result can be attributed to mechanical stresses on wall materials due to drying conditions. Eratte et al. (2014) and Peng et al. (2013) reported the same morphology for microencapsulated powders produced under different drying methods.

### 3.4 Release rate evaluation

**FIGURE 11:**
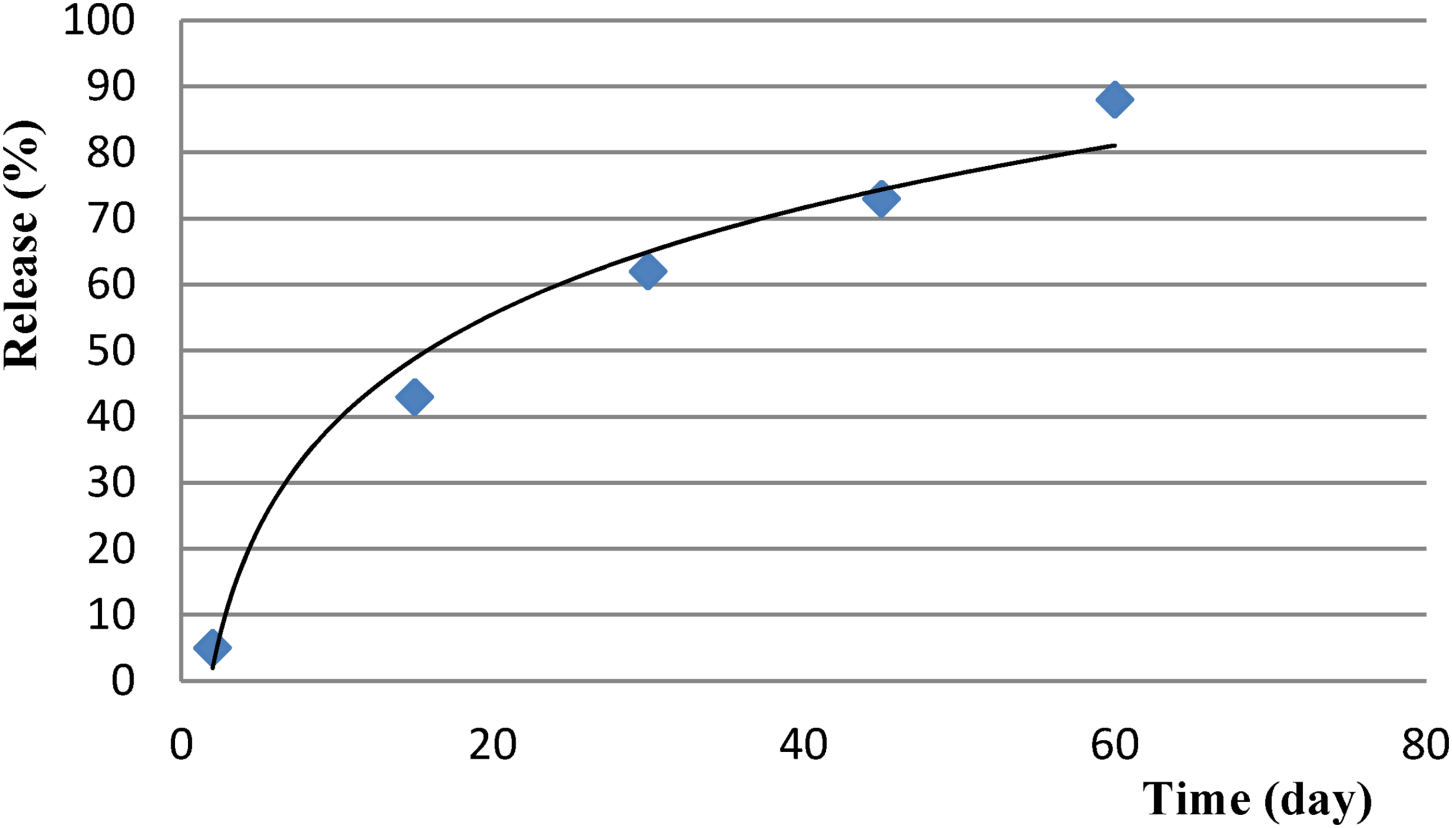
Release of phenolic compounds after 60 days.

According to Figure 11, the release percentage of phenolic compounds reached its maximum value (88%) on the 60th day. The amount of solubility of nanocapsules is one of the most critical characteristics that determines the release rate of substances from the capsules. The increase in substance release rate is due to the decreased stability and cohesion of biopolymers used as coating materials. This leads to a rise in release rates under environmental stresses such as temperature and humidity. Moisture absorption from the environment causes the biopolymer wall to swell. At the same time, its glass transfer temperature decreases. Therefore, coherence and entanglement within polymer chains are reduced, and nanocoated particles exhibit greater molecular mobility. In these conditions, due to the breakdown of the raw materials' texture, compounds entrapped in the nanocapsules will have a higher effective diffusion coefficient (Akbarbaglu et al., 2018; Kalušević et al., 2017).

## 4 CONCLUSIONS

In the present study, an emulsion containing felty germander extract (in concentrations of 10, 20, and 30%) and a mixture of maltodextrin/Persian gum (in three ratios of 1:2, 1:1, and 2:1) as the wall materials were produced and dried in a freeze dryer. Subsequently, the physicochemical properties of the produced emulsion and nanocapsules were investigated. The results showed that the physicochemical properties of emulsions and nanocapsules are affected by the type and concentration of wall materials. The treatments with higher ratios of Persian gum were evaluated as significantly more desirable in terms of all physicochemical properties. In this regard, the optimum physicochemical properties of the produced emulsion and nanocapsules were achieved at a concentration of 10% extract and a 1:1 ratio of maltodextrin/Persian gum as the wall. According to the results, applying maltodextrin and Persian gum carriers and using the freeze dryer can be an effective and promising method to enhance the stability of the nanoencapsulated extract of felty germander in adverse environmental conditions.

## AUTHOR CONTRIBUTIONS

**Sima Zeidvand:** Investigation (equal). **Sara Movahhed:** Conceptualization (equal); supervision (equal). **Hossein Ahmadi Chenarbon:** Formal analysis (equal); software (equal); writing – original draft (equal). **Peyman Rajaei:** Writing – review and editing (equal).

## CONFLICT OF INTEREST STATEMENT

The authors declare that they do not have any conflicts of interest.

## ETHICS STATEMENT

Ethics approval was not required for this research.

## Data Availability

Research data are not shared.
